# Characteristics of e-scooter and bicycle injuries at a university hospital in a large German city – a one-year analysis

**DOI:** 10.1186/s40621-024-00554-w

**Published:** 2025-01-09

**Authors:** Frederik Hartz, Philipp Zehnder, Tobias Resch, Gregor Römmermann, Victoria Hartmann, Markus Schwarz, Chlodwig Kirchhoff, Peter Biberthaler, Michael Zyskowski

**Affiliations:** Department of Trauma Surgery, TUM University Hospital Klinikum Rechts der Isar, Ismaninger Straße 22, 81675 Munich, Germany

**Keywords:** Micromobility, E-scooter, Injury prevention, Emergency medicine, Accident analysis, Head injury, Bicycle

## Abstract

**Background:**

The increasing adoption of individual urban mobility in European cities is contributing to a rise in the number of bicycle and e-scooter users. Consequently, a corresponding increase in accidents, along with an additional burden on emergency departments, is anticipated, particularly in metropolitan areas. The objective of this prospective cross-sectional study was to gather detailed information regarding the patient demographics, accident mechanisms, and injury patterns of e-scooter riders in comparison to cyclists. Identifying any differences between these groups will provide a foundation for developing targeted prevention strategies and safety measures aimed at reducing the incidence of accidents and injuries.

**Methods:**

All patients who presented to the emergency department of our level I university trauma center after an accident involving a traditional bicycle without electric assistance or an e-scooter in 2022 were recorded. Demographic data as well as information regarding the trauma mechanism, injury pattern, alcohol influence, treatment requirements and helmet use were analyzed and compared between the two groups.

**Results:**

In 2022, a total of 626 patients were identified after a bicycle accident and 98 patients after an e-scooter accident. E-scooter riders were with a mean age of 31.0 years (standard deviation (SD) 10.7) significantly younger compared to bicycle riders at 43.2 years (SD 16.5; *p* < 0.001). More than half of the patients in both groups were male (e-scooter 69.4% versus bicycle 60.7%). E-scooter riders were more likely to be intoxicated (31.6% vs. 5.4%; *p* < 0.001), not wearing a helmet (93.9% vs. 78.4%; *p* < 0.001) and to have had accidents at nighttime (39.8% vs. 11.5%; *p* < 0.001). There was no significant difference between the distribution of minor (e-scooter 75.2% vs. bicycle 70.3%) and major (24.8% vs. 29.7%) injuries. In terms of body regions, e-scooter riders suffered from major injuries to the skull, facial cranium, cervical spine (43.8% e-scooter vs. 22.4% bicycle; *p* = 0.008) and less frequently to the trunk, thoracic and lumbar spine and pelvis (0.0% vs. 13.6%).

**Conclusion:**

Compared to cyclists, injured e-scooter riders are younger, mostly do not wear a helmet and more often ride under the influence of alcohol. E-scooter accidents occur more frequently at night and the riders are more likely to suffer serious head injuries.

## Background

Collective health and environmental awareness, the coronavirus pandemic and congested cities have led to modifications regarding transportation in urban areas in recent years, with increasing bicycle use and rapidly growing micromobility (e-scooters, e-bikes, etc.) [1]. In particular, e-scooters which in Germany have been available as part of a sharing rental system since summer 2019, have become increasingly popular for short distance rides with an increasing number of users [2]. Thus, an increase in the number of accidents and presentations in the emergency rooms of inner-city hospitals, resulting in an increasing burden on the healthcare system, is expected [3–5]. Information regarding the population, risk factors, causes of accidents and injury patterns is therefore crucial for initiating preventive measures. Previous studies on e-scooter accidents have shown a significant risk of facial and head injuries, which is likely due to risk factors such as low helmet wearing rates, frequent driving under the influence of alcohol, driving in the dark and biomechanical reasons such as a different driving position and center of body gravity [3, 4, 6–12].

However, studies on the characteristics of e-scooter accidents and accidents involving other means of transportation are still limited and there are only few studies providing detailed information including a comparison between bicycles and e-scooter accidents [9, 13–15].

In this study, we tried to usefully supplement the scientific data by comparing demographic data, mechanisms and aspects of accidents related to injuries caused by riding e-scooters versus bicycles. Furthermore, we provide detailed information on the injury patterns in both groups. This knowledge may help establish transportation-specific prevention measures.

## Methods

### Study design and population

This study enrolled as a prospective cross-sectional study in which all patients who presented after a bicycle or e-scooter accident to our academic level I trauma center in Munich (Germany) during the period of 01.01.2022–31.12.2022 were included. Only bicycles without electric assistance were analyzed in this study. In the case of e-scooters, only standard rental or private electric scooters with a maximum speed of 20 km/h were conducted. At the time of the study, e-scooters could be rented from the companies Tier (now Dott), Voi, Lime and Bolt. Institutional Review Board approval was obtained prior to this study (IRB approval no: 2022-469-S-KK, Ethical Committee of the Technical University Munich).

### Data management and outcomes

Demographic data, accident mechanisms, injury patterns, helmet use, alcohol consumption and therapeutic measures for each group were recorded. Data were collected as part of routine treatment in the emergency room or during hospitalization and were based primarily on information provided by the patient. If the patient was unable to provide information due to the severity of the injury or due to unconsciousness or intoxication, the information from the ambulance or emergency physician protocol was used.

For the purpose of documentation, “night time” was defined as the period between 10:00 pm and 6:00 am and “daytime” as the period between 6:01 am and 09:59 pm, based on the time of the accident. The selected time period is used to distinguish between driving in the dark during leisure time (excluding shift workers) and driving in daylight and during rush hours. The mechanism of the accidents was divided into different categories (evasive maneuvers, collisions with other road users (car, bicycle, e-scooter, pedestrian) or objects (animal, curb, tram rails), slippery road surfaces or individual driving errors without external influence). The injury pattern was categorized by body region into the groups skull/face/neck, trunk (thoracic and lumbar spine, abdomen and pelvis), upper extremity and lower extremity. Furthermore, a distinction was made in all body regions between wounds/soft tissue defects, contusions and sprains, injuries to internal organs and fractures. Wounds, soft tissue defects, contusions, sprains and minor traumatic brain injuries (grade I) were classified as minor injuries. Fractures of the cranium, facial cranium, spine, rib cage or extremities, severe traumatic brain injuries (grade II-III) with or without intracranial hemorrhage and organ injuries in the abdomen were classified as major injuries.

The severity of a traumatic brain injury was classified into levels I-III using the Glasgow Coma Scale (GCS), with mild traumatic brain injury being classified with a GCS of 13–15, moderate with a GCS of 9–13 and severe with a GCS of 3–9 [15]. It was documented if an injury was followed by surgical treatment.

### Comparison

The results of the cyclist group were compared with the e-scooter riders to highlight possible differences. This is particularly important for the effective and targeted implementation of future prevention measures.

### Statistics

Data analysis was performed using Excel (Microsoft Excel V.2018, Microsoft Corporation, Redmond, Washington, USA) and SPSS (IBM Corp. Released 2020. IBM SPSS Statistics for Macintosh Version 27.0. Armonk, NY: IBM Corp). Continuous data were compared with Wilcoxon signed ranks test and reported as mean and standard deviation. Categorical comparisons were analyzed using χ^2^ tests and reported as percentages.

In addition, the correlation between alcohol consumption, helmet wearing rate, mechanism of accident and age was examined for a correlation with major injuries and injuries to certain body regions, such as facial skull fractures or fractures of the lower extremity. The odds ratio (OR) and 95% confidence interval (CI) were stated at this point and χ^2^ or Fisher’s exact test was performed where appropriate. Statistical significance was defined as *p* < 0.05.

## Results

### Demographics and time of accident

A total of 724 patients were detected as eligible for this study and included in the analysis. Among these patients, 626 were treated following a bicycle accident and 98 patients were treated following an accident with an e-scooter. In the bicycle group, 380 (60.7%) patients were male and 246 (39.3%) were female, with a median age of 42 years (range 7–85) and a mean age of 43.2 years (SD 16.5). In the group of e-scooter users, 68 (69.4%) were male and 30 (30.6%) were female, with a median age of 29.5 years (range 17–63) and a mean age of 31 years (SD 10.7).

**Fig. 1:**
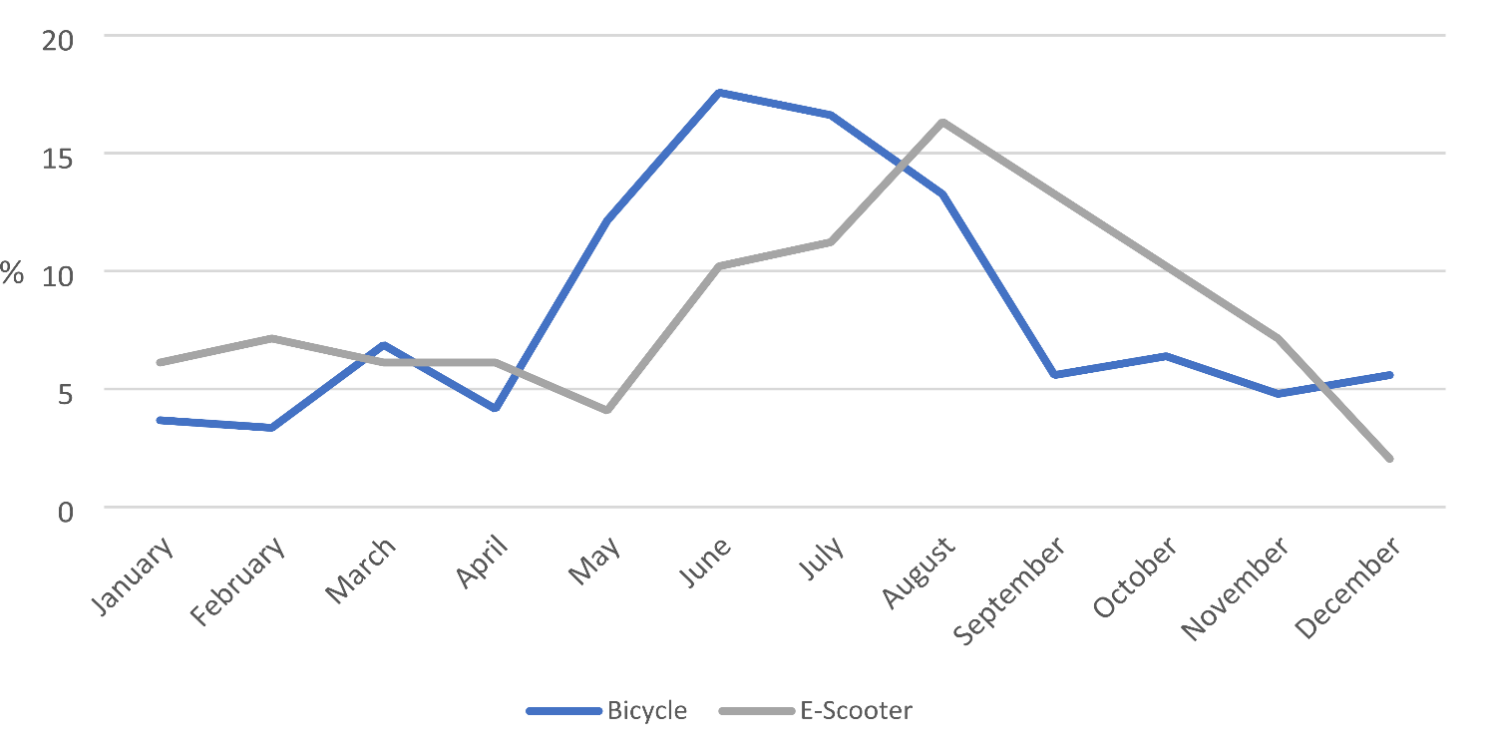
Distribution of accidents by month (in %)

Accidents were recorded throughout the year, with 59.6% of patients presenting after a bicycle accident between May and August. E-scooter users presented particularly frequently from June to October (61.2%). (Fig. 1)

**Fig. 2:**
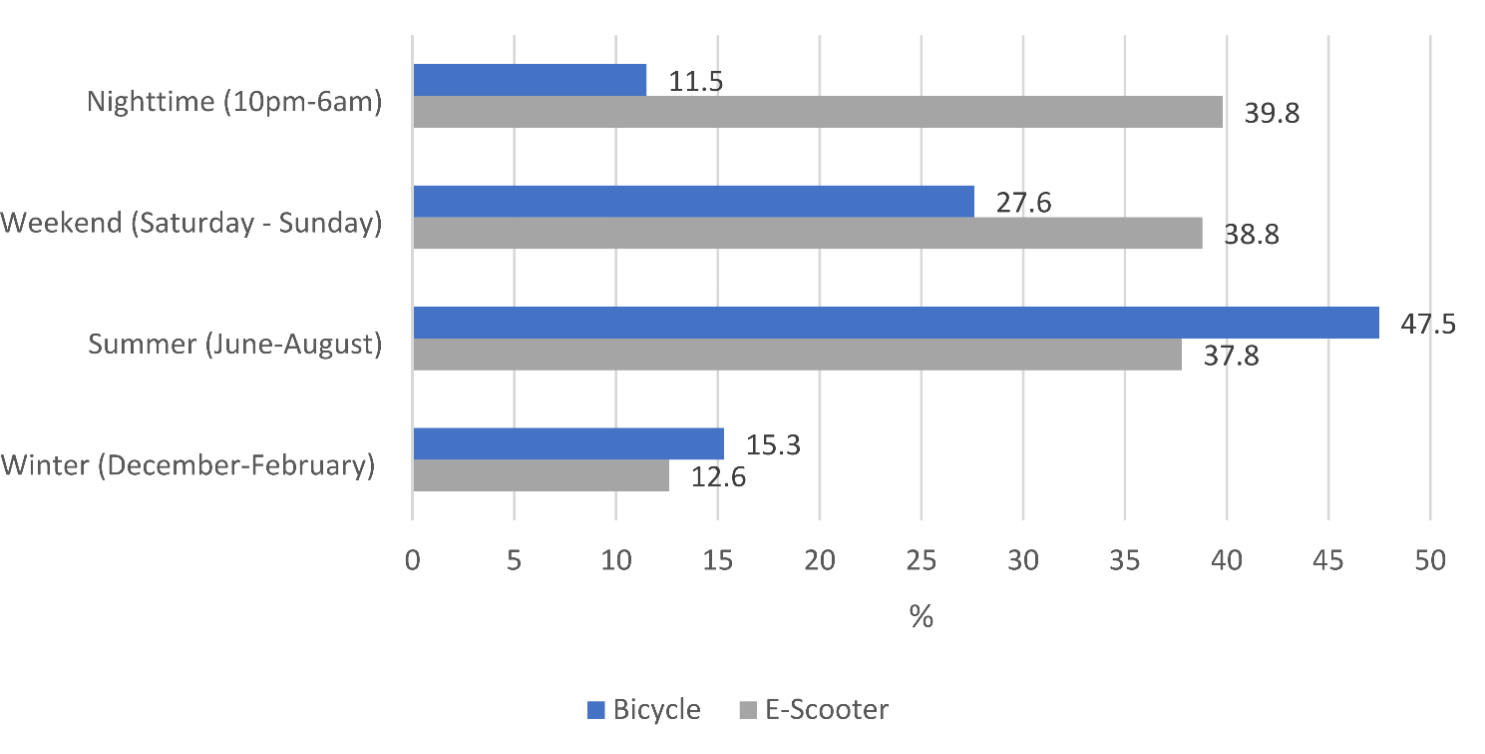
Distribution of accidents by season, weekend and time of day (in %)

In terms of weekdays, cyclist accidents occurred almost evenly distributed. In case of e-scooters, however, more than one in four (26.5%) accidents occurred on Saturdays. Also, there were differences in relation to the time of day. While accidents involving bicycles mainly (77.3%) occurred during daytime (6:01am to 9:59pm) and only 11.5% at night, the difference was considerably smaller for e-scooters, with 39.8% of accidents occurring at night (10pm to 6am). For 70 (11.2%) cyclists and 2 (2.0%) e-scooter riders, the exact time of the accident was unknown. (Fig. 2) No clear trend was identified in terms of the days of the week and the occurrence of major injuries.

More than one in three (34.5%) bicycle accidents were commuting accidents (on the way to or from work), but this only applied to 19.4% of e-scooter users (*p* = 0.003).

### Mechanism of accidents

In 433 (69.2%) out of 626 analyzed bicycle accidents and in 59 (60.2%) out of 98 e-scooter accidents, the cause of the accident was known. In the remaining population the main cause of accidents in both groups was an individual driving error without external influence (e-scooter 28.6%, bicycle 26.3%; *p* = 0.645). Among cyclists another relevant cause of accident was the tires being caught in tram rails, leading to a fall in 7.0% (*n* = 44) of the injured. This mechanism was not detected among the causes of accidents involving e-scooters. In both groups, a common reason for the fall was a slippery road surface (e-scooter 5.1%, bicycle 8.1%; *p* = 0.294). E-scooter riders fell more frequently after a collision with a curb (e-scooter 10.2%, bicycle 2.4%; *p* < 0.001). Collisions with other road users or objects were observed with roughly the same frequency in both groups. (Table 1) In the subgroup analysis, no clear correlation between the mechanism of the accident and the occurrence of major injuries could be detected.

### Transport to the clinic

In both groups, most patients presented themselves to the emergency department on their own without alerting emergency services (e-scooter 80.6%, bicycle 76.8%; *p* = 0.406). Nearly one of five patients in both groups was transported by ambulance (e-scooter 17.3%, bicycle 18.1%; *p* = 0.866). The trauma team was activated with similar frequency in both groups (e-scooter 3.1%, bicycle 5.1%; *p* = 0.379) (Table 1).

### Use of a helmet and alcohol consumption

Significant differences were observed in helmet usage and driving under the influence of alcohol between cyclists and e-scooter riders. Specifically, 21.6% of cyclists wore a helmet, whereas only 4.1% of e-scooter riders did so (*p* < 0.001). Conversely, nearly one-third (31.6%) of e-scooter riders were found to be under the influence of alcohol, compared to only 5.4% of cyclists (*p* < 0.001) (Table 1). A subgroup analysis revealed that e-scooter riders had a significantly higher likelihood of sustaining a major injury following alcohol consumption compared to cyclists. The odds ratio (OR) for alcohol consumption and major injury was 27.342 (95% CI 9.680 to 77.230; Fischer’s exact test *p* < 0.001) for e-scooter riders relative to cyclists.

**Table 1:** Comparison of demographic data and accident characteristics

		Bicycle *n* = 626 (%)	E-scooter *n* = 98 (%)	*p*-value
**Gender**	Male	380 (60.7)	68 (69.4)	0.099
	Female	246 (39.3)	30 (30.6)	
**Age in years**	Mean	43.2	31.0	**< 0.001**
	Standard deviation	16.5	10.7	
**Commuting accident**	Yes	216 (34.5)	19 (19.4)	**0.003**
**Mechanism of accident**	Evasive maneuvers	34 (5.4)	2 (2.0)	0.151
	Slippery road surface	51 (8.1)	5 (5.1)	0.294
	Individual driving error Without external Influence	165 (26.3)	28 (28.6)	0.645
Collision with	Passenger Car	37 (5.9)	5 (5.1)	0.750
	E-scooter	6 (1.0)	–	–
	Bicycle	23 (3.7)	2 (2.0)	0.410
	Pedestrian	8 (1.3)	2 (2.0)	0.547
	Object	41 (6.5)	5 (5.1)	0.585
	Animal	9 (1.4)	–	–
	Curb	15 (2.4)	10 (10.2)	**< 0.001**
	Tram rails	44 (7.0)	–	–
	N/A	193 (30.1)	39 (39.8)	
**Transport to the clinic**	Self-presentation	481 (76.8)	79 (80.6)	0.406
	Via ambulance	113 (18.1)	17 (17.3)	0.866
	Trauma team activation	32 (5.1)	3 (3.1)	0.379
**Use of Helmet**	Yes	135 (21.6)	4 (4.1)	**< 0.001**
	N/A	197 (31.5)	23 (23.5)	
**Alcohol consumption**	Yes	34 (5.4)	31 (31.6)	**< 0.001**
	N/A	146 (23.3)	26 (26.5)	
**Surgery**	Yes	105 (16.8)	10 (10.2)	0.098

Significant *p*-values are printed in bold

### Injury patterns - minor and major injuries

The 626 cyclists suffered a total of 915 injuries, which corresponds to an average of 1.46 injuries per accident. Of the 915 injuries 643 (70.3%) were classified as minor (wounds, bruises, sprains and grade I traumatic brain injury) and 272 (29.7%) as major injuries (grade II and III traumatic brain injury, intracranial hemorrhage, organ injuries, fractures). In the group of e-scooter accidents, 98 riders suffered a total of 129 injuries, which results in an average of 1.31 injuries per accident. Of these 129 injuries, 97 (75.2%) were classified as minor and 32 (24.8%) as major.

**Fig. 3:**
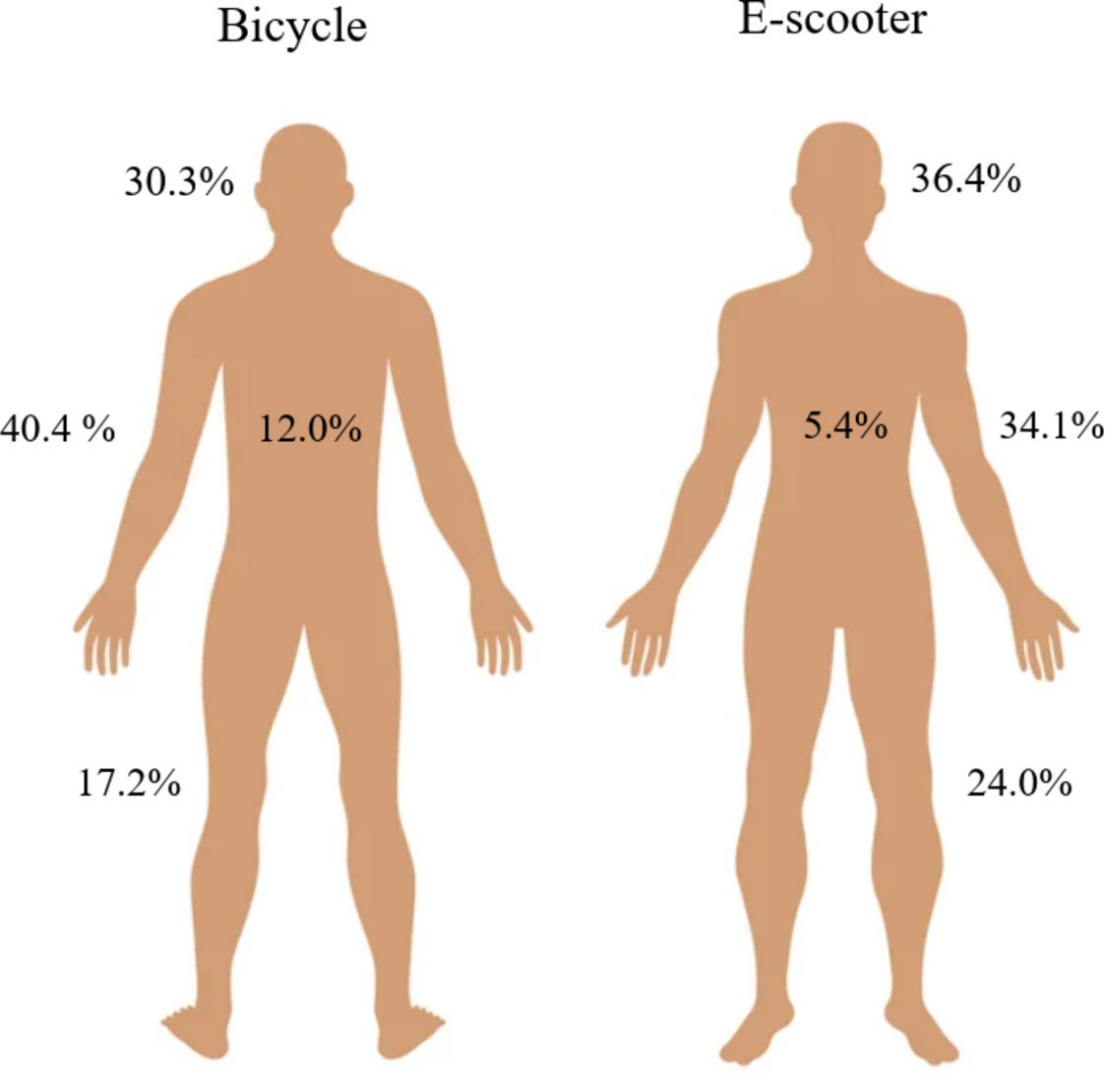
Comparison of the incidence of major and minor injuries combined by body region

In terms of individual body regions, e-scooter riders were most likely to suffer injuries to the head/face or cervical spine (e-scooter 36.4%, bicycle 30.3%; *p* = 0.157). In contrast, the upper extremity was most frequently affected in cyclists (e-scooter 34.1%, bicycle 40.4%; *p* = 0.169). The lower extremity accounted for less than a quarter of injuries for both means of transportation (e-scooter 24.0%, bicycle 17.2%; *p* = 0.062). There was a significant difference in injuries to the trunk, thoracic and lumbar spine and pelvis, which were diagnosed significantly more often in cyclists (e-scooter 5.4%, bicycle 12.0%; *p* = 0.026). (Fig. 3)

### Analysis of major injuries in cyclists and e-scooter riders

A total of 272 major injuries were identified among cyclists, and 32 major injuries among e-scooter riders. Major head and neck injuries occurred significantly more frequently in e-scooter riders (*n* = 14, 43.8%) compared to cyclists (*n* = 61, 22.4%; *p* = 0.008) (Table 2). The most common injury in the head and neck region in both groups was facial cranium fractures (e-scooter: *n* = 7, 21.9%; bicycle: *n* = 30, 11.0%; *p* = 0.076). Among the 30 facial fractures observed in cyclists, only 10.0% (*n* = 3) occurred in helmeted cyclists, while 76.7% (*n* = 23) occurred in cyclists not wearing a helmet. For the remaining cases, helmet use data was unavailable. The odds ratio (OR) for facial cranium fractures in cyclists who wore a helmet was 0.286 (95% CI 0.084 to 0.974; Fisher’s exact test *p* = 0.043), indicating a significant reduction in the likelihood of sustaining a facial fracture in helmeted cyclists. In the e-scooter group, all seven facial cranium fractures occurred in riders not wearing a helmet.

Upper extremity injuries accounted for at least half of the major injuries in both groups (e-scooter: *n* = 16, 50%; bicycle: *n* = 146, 53.7%; *p* = 0.693). The most frequent injury in both groups was a radius head fracture (e-scooter: *n* = 5, 15.6%; bicycle: *n* = 43, 15.8%; *p* = 0.978). Cyclists also sustained major injuries to the trunk, chest, lumbar spine, or pelvis (*n* = 37, 13.6%), with rib fractures being the most common injury (*n* = 27, 10.0%). In contrast, no major trunk or pelvic injuries were observed in e-scooter riders (Table 2).

Lower extremity injuries were relatively rare in both groups (e-scooter: *n* = 2, 6.3%; bicycle: *n* = 28, 10.3%; *p* = 0.468). However, when stratified by age group (≥ 70 years and < 70 years), the analysis revealed an increased likelihood of lower extremity fractures in cyclists aged ≥ 70 years, with an OR of 3.221 (95% CI 1.160 to 8.945; Fisher’s exact test *p* = 0.036). This suggests a higher probability of lower extremity fractures in older cyclists.

**Table 2:** Comparison of major injuries between cyclists and e-scooter riders

Body region	Injury specifics	Bicycle major injuries *n* = 272 (%)	E-scooter major injuries *n* = 32 (%)		*p*-value
**Cranium, facial cranium, cervical spine**		61 (22.4)	14 (43.8)	**0.008**	
	Fracture of the cranium	5 (1.8)	2 (6.3)	0.116	
	Intracranial hemorrhage (SDH, ICH, SAH)	10 (3.7)	2 (6.3)	0.479	
	Facial cranium fracture	30 (11.0)	7 (21.9)	0.076	
	Cervical spine fracture	4 (1.5)	–	–	
	Traumatic brain injury GCS (grad II)	5 (1.8)	1 (3.1)	0.138	
	Traumatic brain injury GCS (grad III)	7 (2.6)	2 (6.3)	0.246	
**Body trunk, chest and lumbar spine, pelvis**		37 (13.6)	–	–	
	Fracture chest and lumbar spine	5 (1.8)	–	–	
	Fracture pelvis	3 (1.1)	–	–	
	Organ injury abdomen/pelvis	2 (0.7)	–	–	
	Rib fracture (≤ 2)	17 (6.3)	–	–	
	Rib fracture (≥ 3)	10 (3.7)	–	–	
**Upper extremity**		146 (53.7)	16 (50.0)	0.693	
Shoulder region	Humeral head fracture	12 (4.4)	–	–	
	Clavicula fracture	23 (8.5)	2 (6.3)	0.667	
	AC-joint dislocation	14 (5.1)	3 (9.4)	0.325	
Elbow	Radial head fracture	43 (15.8)	5 (15.6)	0.978	
	Olecranon fracture	11 (4.0)	–	–	
	Monteggia-like-lesion	2 (0.7)	–	–	
Wrist	Distal radial fracture	16 (5.9)	2 (6.3)	0.934	
Hand and finger	Carpal, metacarpal and finger fracture	25 (9.2)	4 (12.5)	0.547	
**Lower Extremity**		28 (10.3)	2 (6.3)	0.468	
Hip	Femoral neck fracture	4 (1.5)	–	–	
	Pertrochanteric femoral fracture	2 (0.7)	–	–	
Knee	Patella and tibial head fractures	11 (4.0)	–	–	
Ankle	Ankle fracture	6 (2.2)	–	–	
Foot	Foot fracture	5 (1.8)	2 (6.3)	0.116	

significant *p*-value is printed in bold

SDH: subdural hematoma, ICH: intracranial hemorrhage, SAH: subarachnoid hemorrhage, GCS: Glasgow Coma Scale

### Surgical treatment

Among the 626 cyclists involved in accidents, 105 (16.8%) underwent surgical treatment as well as 10 (10.2%) patients of the e-scooter group required surgery (*p* = 0.098), respectively. (Table 1)

## Discussion

This prospective single-center study compared injuries associated with e-scooter and bicycle accidents over a one-year period in an urban area with over half a million people who commute to work daily and a highly developed road and public transportation network [16]. The two modes of transportation showed significant differences in terms of users, injury patterns, and time of accident. One of the key findings was that a higher proportion of serious head injuries were sustained by e-scooter riders (43.8%) than by cyclists (22.4%). The group of cyclists covered almost the entire age spectrum from 7 to 85 years, while the e-scooter riders were mostly between 18 and 40 years old and on average 12 years younger. The young age of e-scooter riders is consistent with the 2023 annual police report in Germany, in which 42% of e-scooter riders involved in accidents were younger than 25 years [5].

The injured patients in both groups of this study were predominantly male (e-scooter 69.4% vs. bicycle 60.7%). The time of accident was for e-scooter riders more often at night (39.8% vs. bicycle 11.5%) and on weekends (38.5% vs. 27.6%). A study by Kleinertz et al. showed similar results for e-scooters (37%) and cyclists (14%) and other studies have also recorded an increased number of e-scooter accidents at night and at weekends [11, 14, 17, 18].

In contrast, the proportion of commuting accidents among cyclists was significantly higher (34.5% vs. e-scooter 19.4%). This could be a result due to a different user profile and mode of use. Compared to bicycles, e-scooters seem to be used mainly by younger people outside of working hours, especially at weekends and at night. In line with this, almost one in three (31.6%) e-scooter riders in this study reported having consumed alcohol, which is consistent with the high proportions (28–71%) reported in other studies [4, 11, 14, 17, 19–21].

The main cause of accident cited by riders in both groups was an individual riding error without external influence. Collisions, particularly with the curb, were also a frequent cause of accidents for e-scooter riders. This goes in line with the results of Meyer et al. who also detected the curb as main obstacle and cause of accident for e-scooter riders [22]. The high proportion of drivers under the influence of alcohol, the lack of driving experience, and driving at night could be risk factors for avoidable driving errors as a cause of accidents [23]. In contrast, a frequent cause of accidents in the bicycle group was tires becoming wedged in the tram rails, which was also described by other authors [22, 24]. Particularly in the city where this study was conducted, which has a dense tram network with 173 stops along 82 km of track, the expansion of bike paths, especially on streets with tram tracks, could counteract this problem [25].

Only 4.1% of e-scooter accident victims wore a helmet. This low rate is consistent with results of other studies [6, 8, 11, 14, 17, 20–22] and in our opinion often due to spontaneous rental use of e-scooters in conjunction with the lack of availability of a helmet at the rental station. Even though the helmet wearing rate among cyclists in this study was significantly higher at 31.5%, the majority of injured did not wear a helmet. While the protective effect of helmets on e-scooters has not yet been scientifically investigated, previous studies have shown that wearing a helmet while cycling reduces the risk of traumatic brain injury [26–29]. In this study, wearing a helmet reduced the likelihood for facial cranium fractures in cyclists (OR 0.286, 95% CI 0.084 to 0.974; *p* = 0.043). Although the value was only just above the significance level, Bellal et al. were also able to show in their study that cyclists who wore a helmet had a 31% lower probability of facial fractures (OR 0.69, 95% CI 0.58 to 0.81, *p* < 0.001) and severe traumatic brain injury (OR 0.49, 95% CI 0.43 to 0.55) [30]. Based on these results, a protective effect for wearing a helmet could also be expected for e-scooter riders.

In addition to awareness-raising campaigns, the availability of helmets in the e-scooter hire system should therefore be considered. In Australia, Haworth et al. were able to show that an available helmet when renting an e-scooter could increase the helmet wearing rate to up to 64% [31].

In both groups, the majority presented themselves on their own to the emergency department with mainly minor injuries. Overall, the head/face/neck region (36.4%) is predominantly affected in e-scooter injuries, followed by the upper (34.1%) and finally the lower extremities (24.0%). Injuries to the lower extremities are almost exclusively minor injuries such as wounds, bruises or sprains. Frequent soft tissue injuries to the ankle in e-scooter riders were also described in the study by Uluk et al. [10]. Among the cyclists involved in accidents, the upper extremities (40.4%) were the most frequently affected, followed by the head/face/neck region (30.3%) and the lower extremities (17.2%).

In this study, there were significant differences in the distribution of major injuries across the body regions between the two modes of transportation.

The proportion of major head and facial injuries was significantly higher for e-scooter riders (43.8%) than for cyclists (22.4%). Fractures of the facial skull in particular were frequently diagnosed at 21.9%. In their study, Grill et al. also reported more facial injuries in e-scooter accident victims than in cyclists [6]. Possible reasons for this could be factors such as a lower helmet-wearing rate and driving under the influence of alcohol. In general, e-scooter riders in this study who had consumed alcohol were significantly more likely to suffer major injuries than cyclists (OR 27,342, CI 9,680 to 77,230; *p* < 0.001). It is also likely that the accident kinematics differ between the two means of transportation. In its simulation study of e-scooter accidents, the Fraunhofer Institute for High-Speed Dynamics, Ernst-Mach-Institute, was able to show that, depending on the impact speed and angle on an obstacle such as a curb, the e-scooter can act on the rider like a lever and catapult them into the air due to the e-scooter’s mass inertia [12]. This results in a higher force on the upper extremities and the face/head on impact, which can lead to potentially major injuries. Further biomechanical accident analyses could contribute to a better understanding of accident kinematics and provide insights into possible preventive measures.

In this study the upper extremity was similarly affected by major injuries (e-scooter 50.0% vs. bicycle 53.7%). In a review by Luceri et al. the upper limbs were also most frequently affected by fractures in e-scooters [32]. However, injuries to the trunk of the body such as rib cage fractures (10.0%) and fractures of the lower extremities (10.3%) such as hip or knee joint-associated fractures (2.2%; 4.0%) were only observed in cyclists and did not play a role in e-scooter accidents in this study. Additionally, the analysis of fractures of the lower extremities in cyclists showed an increased probability in riders aged ≥ 70 years (OR 3.221, CI 1.160 to 8.945, *p* = 0.036). The previously mentioned kinematics of e-scooter falls and the higher average age of cyclists could be a reason for the occurrence of osteoporotic fractures such as proximal femur or hip fractures.

Overall, general accident prevention is likely to have the greatest influence on the frequency of injuries and should be the primary goal of future prevention measures. General measures such as the improvement of infrastructure and transport-specific prevention programs seem to be necessary. Specific measures to reduce the risk of injury for e-scooter riders should primarily target the young users, who are the most affected population [14]. Some providers in Germany, such as Tier (now Dott) and Voi, have implemented an alcohol reaction test in their app before renting. However, the effect and reliability have not yet been validated in studies. In their study, Pakarinen et al. were able to show that regional and temporal (especially at night and on weekends) reduction in driving speed could also significantly reduce the number of accidents and recommend the measures for other major cities [23]. Additionally, the implementation of a ‘rookie mode’, where first-time e-scooter users initially drive at reduced speed and in daylight to develop driving experience, could help prevent accidents. We also believe that mandatory disclosure of accident data by e-scooter rental companies would help to better identify and mitigate risk periods and risk areas in urban areas.

## Limitations

Several limitations must be considered in the interpretation of our findings. First, this was a single-center study, and the data collection period was limited to one year. Additionally, data on factors such as the mechanism of the accident, helmet use, and alcohol consumption were primarily obtained from patient self-reports, which introduces the potential for recall bias or inaccurate reporting. In particular, the data on accident mechanisms and alcohol consumption contained a substantial proportion of missing values, likely due to insufficient documentation or incomplete patient information. As a result, some degree of deviation in these variables cannot be excluded, although it is our opinion that alcohol consumption figures are likely to be underestimated in this study.

We believe that the results of this study are generally applicable to other large urban environments. However, certain findings, such as the high frequency of cyclists falling due to tires becoming wedged in tram rails, may be specific to cities with active tram systems.

In terms of injury classification, we opted not to use a standardized injury severity score (e.g., ISS), but instead chose to categorize each injury as either minor or major. This approach allows for a more detailed analysis of specific injury patterns within both groups, although it may limit the comparability of overall injury severity across studies.

## Conclusion

Significant differences were observed between e-scooter riders and cyclists with respect to patient demographics, usage and injury patterns. E-scooter accidents predominantly involve young male riders, occurring primarily on weekends and during nighttime, often with alcohol consumption as a contributing factor. In contrast, bicycle accidents affect a wider range of age groups, with over one-third of incidents being related to commuting. Head and upper limb injuries were common in both groups, but the incidence of severe head and facial injuries was particularly concerning among e-scooter riders. Furthermore, the rate of helmet use was significantly lower among e-scooter riders compared to cyclists.

Based on these findings, several preventive measures may prove beneficial. These include targeted awareness campaigns for young e-scooter riders, regulation of e-scooter availability and speed during high-risk periods (weekends and nighttime), improved access to helmets, and stricter enforcement of alcohol-related regulations for e-scooter riders.

## Data Availability

The datasets used and/or analyzed during the current study are available from the corresponding author on reasonable request.

## Abbreviations

SD: Standard deviation
vs.: Versus
am: Ante meridiem
pm: Post meridiem
N/A: Not applicable
etc.: et cetera
n: Sample size
e.g.: Exempli gratia/for example
SDH: Subdural hematoma
ICH: Intracranial hemorrhage
SAH: Subarachnoid hemorrhage
GCS: Glasgow Coma Scale
OR: Odds ratio
CI: Confidence interval
